# Association of adenosine signaling gene signature with estrogen receptor-positive breast and prostate cancer bone metastasis

**DOI:** 10.3389/fmed.2022.965429

**Published:** 2022-09-15

**Authors:** Daniel Brian Shropshire, Francisca M. Acosta, Kun Fang, Jaime Benavides, Lu-Zhe Sun, Victor X. Jin, Jean X. Jiang

**Affiliations:** ^1^Department of Biochemistry and Structural Biology, University of Texas Health Science Center, San Antonio, TX, United States; ^2^Division of Biostatistics and MCW Cancer Center, Medical College of Wisconsin, Milwaukee, WI, United States; ^3^Department of Biomedical Engineering and Chemical Engineering, The University of Texas at San Antonio, San Antonio, TX, United States; ^4^Department of Cell Systems and Anatomy, University of Texas Health Science Center, San Antonio, TX, United States

**Keywords:** metastasis, bone, breast, prostate, purinergic, osteocyte

## Abstract

Bone metastasis is a common and devastating consequence of several major cancer types, including breast and prostate. Osteocytes are the predominant bone cell, and through connexin (Cx) 43 hemichannels release ATP to the bone microenvironment that can be hydrolyzed to adenosine. Here, we investigated how genes related to ATP paracrine signaling are involved in two common bone-metastasizing malignancies, estrogen receptor positive (ER^+^) breast and prostate cancers. Compared to other sites, bone metastases of both cancer types expressed higher levels of ENTPD1 and NT5E, which encode CD39 and CD73, respectively, and hydrolyze ATP to adenosine. ADORA3, encoding the adenosine A3 receptor, had a similar expression pattern. In primary ER^+^ breast cancer, high levels of the triplet ENTPD1/NT5E/ADORA3 expression signature was correlated with lower overall, distant metastasis-free, and progression-free survival. In ER^+^ bone metastasis biopsies, this expression signature is associated with lower survival. This expression signature was also higher in bone-metastasizing primary prostate cancers than in those that caused other tumor events or did not lead to progressive disease. In 3D culture, a non-hydrolyzable ATP analog inhibited the growth of breast and prostate cancer cell lines more than ATP did. A3 inhibition also reduced spheroid growth. Large-scale screens by the Drug Repurposing Hub found ER^+^ breast cancer cell lines were uniquely sensitive to adenosine receptor antagonists. Together, these data suggest a vital role for extracellular ATP degradation and adenosine receptor signaling in cancer bone metastasis, and this study provides potential diagnostic means for bone metastasis and specific targets for treatment and prevention.

## Introduction

Bone is the most common site for distant metastasis by breast and prostate cancers and has devastating impacts on patients (1, 2). Complications include severe pain, pathologic fractures, life-threatening hypercalcemia, and spinal cord compression (3, 4). Furthermore, patients with bone metastases have poor overall prognosis and lower life expectancies (5–7). Understanding the process that permits breast and prostate cancer bone metastasis and knowing how to derail it is critical for improving patient outcomes for the second-leading cause of cancer deaths in women and men, respectively. The microenvironment of distant organs plays a vital role in the process of metastasis to that site (8). Despite this, few drugs specifically target metastatic sites. Bisphosphonates induce osteoclast apoptosis, promote osteocyte Cx43 hemichannel activity (9, 10), and are used to treat bone metastases of various types, including prostate and breast (11, 12). More uniquely, they were clinically validated to prevent breast cancer metastasis to bone in postmenopausal women (13).

Osteocytes comprise roughly 90% of bone cells and are dominant regulators of the local microenvironment (14). In normal bone physiology, they coordinate the actions of bone-building osteoblasts and bone-degrading osteoclasts (14). Osteocytes are rich in Cx43 hemichannels, through which small paracrine signaling molecules such as prostaglandins and ATP are released and influence both normal bone cells and metastatic cancer cells (15, 16). Our previous study found osteocytes expressing Cx43 with impaired hemichannel and gap junction activity promoted the growth of triple-negative breast cancer in bone, while osteocytes with impaired Cx43 gap junction but retained hemichannel function had no such effect (17). Further investigation showed that a stable extracellular ATP (eATP) analog decreased triple-negative breast cancer cell migration, while extracellular adenosine (eADO) increased it, and thus preventing eATP degradation to eADO can enhance the inhibitory effect of eATP on cancer cell migration (16).

A recent surge of interest in purinergic signaling in cancer is primarily on its role in immunology. In tumors, eATP is elevated and generally stimulates the immune system (18). This eATP can be hydrolyzed to AMP by CD39, encoded by the gene ENTPD1, and further degraded to adenosine by CD73, encoded by the gene NT5E (18). The immunosuppressive function of eADO is in part mediated by binding to T cell adenosine 2A receptors (A2ARs) (18). This rationale has led to interest in inhibiting eADO production in tumors as a way of improving outcomes alone or combined with PD1-PDL1 inhibition (19, 20). However, much less attention has been given to the non-immunologic functions of eATP and eADO in cancer development and progression. Our studies on ATP release by osteocytic hemichannels in bone and the effects of eATP and eADO signaling on triple-negative breast cancer led us to investigate whether tumor cells increase eATP hydrolysis to promote bone metastasis. We focused on estrogen receptor-positive (ER^+^) breast cancer, which accounts for 77% of breast cancer bone metastases (1), and prostate cancer, which also primarily metastasizes to bone (2, 7).

## Materials and methods

### Materials

Spheroid culture plates were purchased from Corning (Corning, NY, United States; cat. 4515). ATP was purchased from Sigma Aldrich (St. Louis, MO, United States; cat. A2383). ATPγS was purchased from Fisher (Hampton, NH, United States; cat. 40-801-0). Both were dissolved in Dulbecco’s phosphate buffered saline (Gibco cat. 14190). MRS-1220 (cat. 12-175) was purchased from Fisher and dissolved in dimethyl sulfoxide (Fisher cat. 67-68-5). The rest of the reagents were purchased either from Fisher or Sigma.

### Cell culture, 3D culture, and quantification

MCF-7 cells were a gift from Dr. Michael Brattain maintained in Dulbecco’s Modification of Eagle’s Medium (DMEM) with 10% fetal bovine serum (FBS). 22Rv1 cells were a gift from Dr. Tim Huang at University of Texas Health Science Center at San Antonio and were maintained in RPMI-1640 with 10% FBS. Cells were kept in a 5% CO_2_ incubator.

For 3D culture, 2,000 cells per well were seeded in ultra-low adherent U-bottom 96-well plates with drug or vehicle in DMEM with 2.5% FBS (MCF-7) or RPMI-1640 with 2.5% FBS (22Rv1). Photos were taken using a Keyence BZ-X710 microscope (Keyence, Osaka, Japan) using a 20X phase contrast objective (Nikon, Tokyo, Japan). Sphere cross-sectional area was measured using ImageJ, (21) which was used to determine volume. Statistical comparisons were made using *t*-test or two-way ANOVA with the Geisser-Greenhouse correction and Tukey’s post-test. EC50 values were calculated in Graphpad Prism v9 using a four-parameter logistical model.

### Ribonucleic acid expression in metastases, and comparison with primary tumor

Microarray datasets GSE74685, GSE14020, GSE32269, and GSE47561 were downloaded from the Gene Expression Omnibus. GSE14020 raw fluorescence CEL files were processed using BART (22). Datasets were chosen based on clinical characteristics (Supplementary Table 1), using workflow as shown in Supplementary Figure 1. Differential gene expression analysis for Supplementary Table 2 was performed using the limma bioinformatics package (23). We compared log2-transformed data in metastatic locations containing at least 5 samples using one-way ANOVA and Dunnett’s multiple comparisons test. Expression between primary and metastatic tumors was compared using a *t*-test. For breast cancer, the Robust Microchip Array (RMA) function in Bioconductor was used to process primary and metastatic data.

### Survival analysis

**FIGURE 1:**
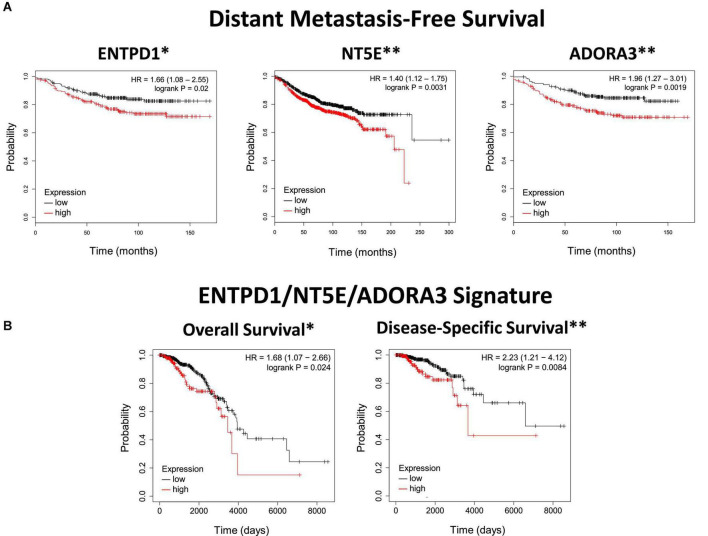
ENTPD1, NT5E, and ADORA3 expression in primary ER^+^ breast cancer correlates with poor outcomes. **(A)** Kaplan Meier plots of distant metastasis-free survival in ER^+^ breast cancer. The high-expression groups for ENTPD1 (HR = 1.66), NT5E (HR = 1.4), and ADORA3 (HR = 1.96) all have a significantly greater chance of distant metastasis or death. Analysis was made using KMPlot **(24)**. **(B)** Kaplan Meier plots of overall and disease-specific survival of ER^+^ breast cancer patients in the TCGA BRCA cohort based on ENTPD1/NT5E/ADORA3 signature expression (calculated by the average expression [log2(norm_count + 1)] of the three genes, and separated by median). In this separate cohort than **(A)**, the high-expression group had significantly lower overall and disease-specific survival (HR = 1.68 and 2.23, respectively). *p < 0.05; ^**^p < 0.01.

**FIGURE 2:**
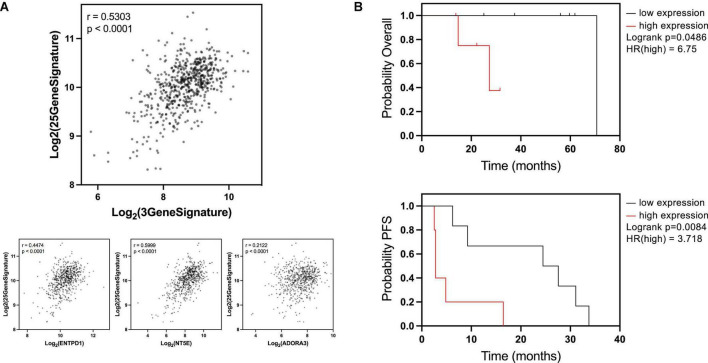
ENTPD1/NT5E/ADORA3 signature is correlated with breast cancer osteotropic signature and lower survival in bone patients with established bone metastases. **(A)** A previous study (28) found 25 genes to be upregulated in circulating breast cancer cells from patients with bone metastases compared to patients with extraskeletal metastases, forming a putative bone metastasis-specific signature. We compared ENTPD1/NT5E/ADORA3 expression signature to the 25-gene osteotropic signature in the ER^+^ TCGA BRCA cohort. A strong Pearson correlation (*r* = 0.5303) was observed. Individually, ENTPD1, NT5E, and ADORA3 were each correlated *r* > 0.2. These data imply that the 3-gene signature is not an overall metastasis marker in ER^+^ breast cancer and is specifically associated with bone metastasis. **(B)** Kaplan Meier plots displaying overall and progression-free survival in patients with ER^+^ breast cancer bone metastasis. In GSE124647, gene expression was measured in bone biopsy samples. We split this cohort into high- and low-expressing ENTPD1/NT5E/ADORA3 signature (calculated by the average expression [log2(norm_count + 1)] of the three genes, and separated by median). In this *n* = 13 cohort, high 3-gene signature expression was associated with lower overall (HR = 6.75) and progression-free (HR = 3.718) survival, further suggesting ectonucleotidase and ADORA3 expression enables breast cancer growth in the bone microenvironment. *p < 0.05; **p < 0.01; ****p < 0.0001.

Distant metastasis-free survival analysis in ER^+^ breast cancers was performed on microarray data using KMPlot (24, 25). We used ER^+^ patients because the first distant metastasis in these patients is usually located in bone (1). Expression data was used to predict ER status when not histologically determined. Patients were separated into high- and low-expressing tumors by median, as evenly as possible. Overall and disease-specific survival were performed using data from the TCGA BRCA (26) cohort accessed through Xena browser (27) and analyzed through KMPlot (24). Signatures were calculated by the average expression [log_2_(norm_count + 1)] of the three genes when noted in Figures 1B, 2. Survival analysis for samples taken from established bone metastases used GSE124647 and cohorts were separated by median expression. Significance was determined by *p* < 0.05.

### Gene signature and Gleason score correlation

Signature correlation was performed on ER^+^ tumors in the TCGA BRCA cohort using a previously published gene set (26, 28). TCGA PRAD (29) data (counts) were downloaded through Xena browser (27). DKFZ data (counts) were downloaded from cBio Cancer Genetics Portal (30, 31). Expression signatures were the average expression of the three genes in each sample. Pearson method was used for correlation analysis. One-way ANOVA with a test for linear trend was used to find increasing averages with increasing Gleason scores and Kruskall-Wallis test with multiple comparisons for comparing signature expression between primary tumors with or without bone metastases and other events. Significance was determined by *p* < 0.05.

### Drug sensitivity determination

Drug screen was performed using PRISM technique (32) by the Drug Repurposing Hub, as reported (33). Analyses were performed on the 19Q3 screen. Data were analyzed on DepMap (34) portal, which uses the Limma R statistical package (23). Significance was determined by *p* < 0.0005.

### Statistics

Statistical analyses were performed on Graphpad Prism v9 unless otherwise noted. Graphs reflect mean ± SD, except DepMap screen in which boxes represent median ± 1 interquartile range and whiskers represent 5th and 95th percentiles. *p < 0.05; ^**^p < 0.01, ^***^p < 0.001, ^****^p < 0.0001, except for DepMap screen where *p* < 0.0005 is significant.

## Results

### ENTPD1, NT5E, and ADORA3 show higher expression in bone metastases than in other sites of metastasis or in primary tumors

**FIGURE 3:**
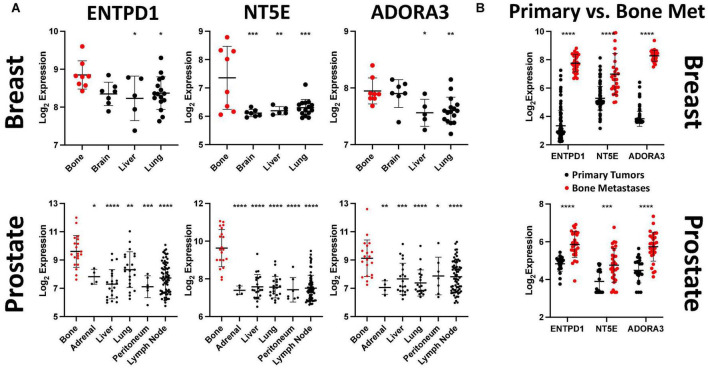
ENTPD1, NT5E, and ADORA3 expression are much higher in bone metastases than in other metastases or primary tumors. **(A)** Relative expression of ENTPD1, NT5E, and ADORA3 in metastatic breast and prostate tumors in various organs in GEO datasets GSE14020 and GSE74685. We found that ENTPD1, NT5E, and ADORA3 tend to be more highly expressed in breast and prostate cancer bone metastases (red) than in metastases to other sites (black). **(B)** We found significantly higher expression of ENTPD1, NT5E, and ADORA3 in bone metastases (red) than in primary breast (black, GSE47561) or prostate (black, GSE32269) cancers. One-way ANOVA with Dunnett’s post-test was used in **(A)** and unpaired Student’s *t*-test was used in **(B)**. *p < 0.05; **p < 0.01; ***p < 0.001; ****p < 0.0001.

We previously identified ATP released by active hemichannels as a potential inhibitor of triple-negative breast cancer growth in bone (16, 17). Because hemichannels are rare in most tissues but are well established in bone, we hypothesized that downregulating one or more ATP receptors would enable bone metastasis, and this receptor would have lower expression levels in bone metastases compared to metastases at other locations. We investigated this in microarray gene expression data from patients with metastatic breast cancer (GSE14020). Surprisingly, none of the ATP receptors was differentially expressed between bone and other metastatic sites (Supplementary Table 2). However, ENTPD1 and NT5E, which encode genes that degrade eATP to eADO, were more highly expressed in bone metastases than in metastases to other sites (Figure 3A),top. We next investigated which receptors are activated by the excess eADO formed by eATP hydrolysis and found increased expression of ADORA3, encoding A3R, in bone metastases (Figure 3A),top. We also analyzed gene expression in metastatic prostate cancer (GSE74685) and found a similar expression pattern, with ENTPD1, NT5E, and ADORA3 upregulation in bone metastases than in other metastases (Figure 3A), bottom.

After determining that these three genes are more highly expressed in bone metastases than in other metastases, we further compared their expression between bone metastases and primary tumors. ENTPD1, NT5E, and ADORA3 showed higher expression in bone metastases than in primary breast cancers (Figure 3B),top, GSE47561. Similarly, castrate-resistant bone metastases had higher expression of these three genes than did primary prostate cancer (Figure 3B), bottom, GSE32269. Taken together, we demonstrated that the expression of two genes that hydrolyze eATP to eADO and the eADO receptor ADORA3 are more highly expressed in bone metastases than in other metastases or in primary tumors.

### High expression of ENTPD1, NT5E, and ADORA3 in primary ER^+^ breast cancer is correlated with lower distant metastasis-free survival, overall survival, and disease-specific survival

Next, we investigated whether primary tumors with higher expression of these genes are more likely to metastasize to bone. Since bone is the site of first metastasis for the majority of patients with ER^+^ breast cancer, (35) distant metastasis-free survival in these patients should largely reflect bone metastasis. Primary breast cancer microarray expression studies that reported this outcome were normalized and pooled by KMPlot (24). We found the high-expression cohort for each of ENTPD1, NT5E, and ADORA3 had significantly lower distant metastasis-free survival (Figure 1A). None of the other adenosine receptors was significantly correlated with distant metastasis in patients with ER^+^ breast cancer (Supplementary Figure 2A). To further explore how gene expression in primary tumors might be related to prognosis, we analyzed overall and disease-specific survival among those with ER^+^ tumors among the TCGA BRCA cohort. The top half of the 3-gene expression signature (calculated by the average expression [log2(norm_count + 1)] of the three genes, and separated by median) fared more poorly in both outcomes, with an especially strong relationship with disease-specific survival (Figure 1B). Additionally, there was generally a stronger relationship with the signature than each individual gene (Supplementary Figure 2B). The ENTPD1/NT5E/ADORA3 expression signature was not correlated with either outcome in ER^–^, HER2-enriched, or basal breast cancers, which do not share the same metastatic behavior (Supplementary Figure 3A), nor were signatures combining expression of the ectonucleotidases with any of the other aADO receptors in ER^+^ tumors (Supplementary Figure 3B).

Because these outcomes do not measure bone metastasis specifically, we compared the 3-gene signature to an osteotropic breast cancer gene signature (28). To determine this signature, targeted RNA-Seq was performed on circulating cancer cells of patients with metastatic breast cancer. There were 25 genes upregulated in patients with bone metastases compared to patients with extraskeletal metastases. The expression signature combining these 25 genes exhibited a strong correlation with the 3-gene ENTPD1/NT5E/ADORA3 signature in ER^+^ breast cancers in the TCGA BRCA cohort, and this relationship is also observed with each of the three genes individually (Figure 2A). Because these genes are associated with metastasis to bone, but not to other locations, this suggests that our data are specifically reflective of bone metastasis and not of the overall metastatic ability or aggressiveness.

### ENTPD1/NT5E/ADORA3 gene signature in breast cancer bone metastases can predict poor prognosis

If the higher expression of these genes facilitates breast cancer growth in bone, then their elevated expression in already established bone metastases may promote further tumor progression. We compared overall survival and progression-free survival in ER^+^ breast cancer bone metastases based on the median expression of the three-gene signature. Patients whose tumors were above the median expression level had a significantly lower overall survival and progression-free survival than patients below the median expression (Figure 2B). Notably, expression of none of these genes was individually correlated with overall survival (Supplementary Figure 4, top) and only ADORA3 was significantly correlated with progression-free survival (Supplementary Figure 4, bottom). This analysis provides further support for the hypothesis that the eATP to eADO hydrolysis might be directly linked to the process of breast cancer bone metastases and overall survival.

### Higher expression of ENTPD1/NT5E/ADORA3 gene signature in primary prostate cancer is associated with bone metastasis, but not other progression

**FIGURE 4:**
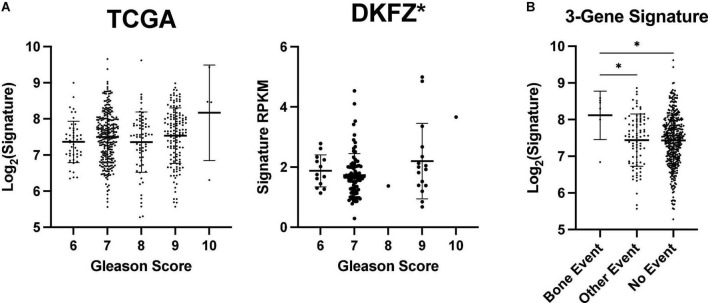
ENTPD1/NT5E/ADORA3 expression level is higher in primary prostate tumors that metastasize to bone. Gleason scores reflect the undifferentiation of prostate tumors. Tumors are given two scores that are often added together such as in TCGA PRAD dataset. Tumors with higher scores are more likely to metastasize to bone and other poor outcomes **(36)**. **(A)** An increasing Gleason score is associated with higher ENTPD1/NT5E/ADORA3 expression signature in the DKFZ but not the TCGA PRAD cohort. **(B)** 3-gene expression signature is higher in tumors that form bone metastases, than in tumors that do not progress or cause other events. **(A)** One-way ANOVA with test for trend, **(B)** Kruskal–Wallis test with multiple comparisons. *p < 0.05.

We first compared their expression levels across Gleason scores, a measure of tumoral undifferentiation where tumors are given two scores for the dominant and non-dominant phenotype that are often combined into one score. Higher Gleason scores are associated with a worse prognosis and a greater likelihood of recurrence, bone metastasis, and mortality (36–38). There was a significant trend of increasing signature expression with increasing Gleason scores in the German Cancer Research Center cohort (Deutsches Krebsforschungszentrum, DKFZ), (39) but not TCGA PRAD cohort (Figure 4A). Thus, the ENTPD1/NT5E/ADORA3 signature does not have a strong relationship with undifferentiation. Notably, most patients who present with localized disease and high Gleason scores do not suffer from bone metastasis in the next 15 years (40). Further, Gleason score and the National Comprehensive Cancer Network combined clinicopathologic score are outperformed by the FDA approved Decipher^®^ Genomic Classifier (41, 42). In the TCGA PRAD cohort, bone-event-causing primary tumors had higher 3-gene signature expression than did those that did not progress, and those that caused other new tumor events, which in this cohort comprise biochemical recurrence, new primary tumor, locoregional metastasis, and distant metastasis to other locations (Figure 4B). This suggests that the ENTPD1/NT5E/ADORA3 expression signature is specific for bone metastasis and not of other disease progressions.

### Extracellular ATP and A3R antagonist MRS-1220 inhibit breast and prostate cancer cell growth in 3D culture, and non-hydrolyzable ATP analog ATPγS causes stronger reduction

**FIGURE 5:**
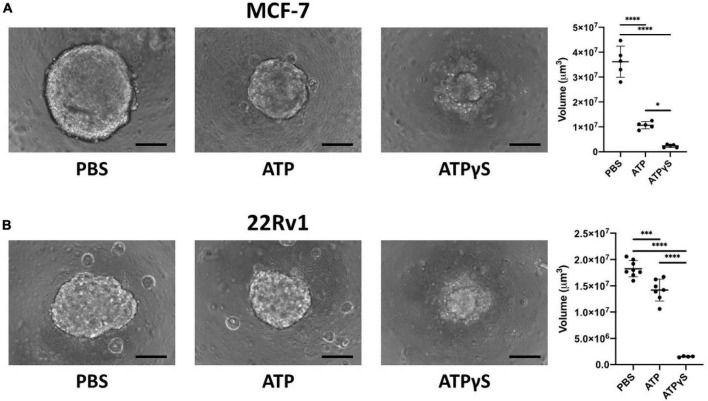
Extracellular ATP (eATP) inhibits MCF-7 and 22Rv1 breast cancer cell growth in 3D culture and non-hydrolyzable ATP analog ATPγS causes stronger reduction. We cultured 2000 MCF-7 **(A)** or 22Rv1 **(B)** cells in 3D culture conditions for 1 week with 1mM ATP, ATPγS or vehicle before acquiring images using a Keyence BZ-X710 microscope and determining sphere volume. ATP significantly inhibited the sphere size compared to vehicle, and its non-hydrolyzable analog significantly further reduced sphere size. One-way ANOVA with Tukey’s post-test was used for pairwise comparisons. *p < 0.05; ^***^p < 0.001; ^****^p < 0.0001. Scale bar = 200 μm.

**FIGURE 6:**
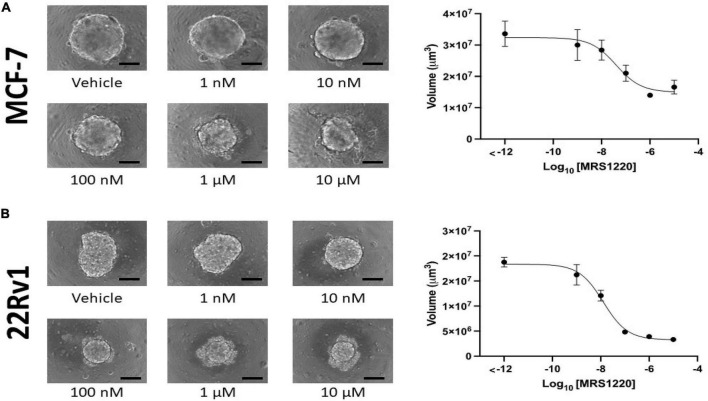
Adenosine A3 receptor antagonist MRS-1220 inhibits MCF-7 and 22Rv1 cells in a dose-dependent manner. We incubated 2000 MCF-7 **(A)** and 22Rv1 **(B)** cells in 3D culture conditions for 1 week before determining sphere volume. A dose-dependent inhibition was observed in both cell lines, with EC50 values of 39 nM (95% CI 11.27–110 nM) in MCF-7 cells and 13 nM (95% CI = 8.629–18.39 nM) in 22Rv1 cells. Scale bar = 200 μm.

We next used relevant *in vitro* models to determine whether these data reflect a confounding variable or if higher expression of these genes may facilitate bone metastasis. We first compared the effects of ATP and its non-hydrolyzable analog ATPγS on MCF-7, (43) an ER^+^ breast cancer cell line and 22Rv1, (44) a prostate cancer cell line that originated from the primary tumor of a patient with bone metastasis (45) and that generates mixed osteoblastic and osteolytic tumors in bone (46). In 3D culture conditions ATPγS strongly inhibited growth of MCF-7 cells compared to ATP, which is subject to hydrolysis by CD39 and CD73 encoded by ENTPD1 and NT5E genes, respectively. Both conditions inhibited growth compared to PBS vehicle control (Figure 5A). Similar results were obtained in 22Rv1 cells (Figure 5B). These data show that eATP signaling inhibits the growth of breast and prostate cancer cells and that eATP hydrolysis is a mechanism that averts these effects. We also investigated how inhibition of A3R, encoded by the ADORA3 gene, affects growth in 3D culture using MRS-1220, a specific A3R inhibitor. We found dose-dependent growth inhibition in both MCF-7 and 22Rv1 cells in 3D culture with an EC50 of 39 nM in MCF-7 cells and 13 nM in 22Rv1 cells (Figure 6). Together, these data demonstrate the importance of eATP hydrolysis and the reliance on A3 signaling for ER^+^ breast cancer and prostate cancer cells.

### ER^+^ breast cancer cell lines are uniquely sensitive to non-xanthine adenosine receptor antagonists in the drug repurposing Hub

**FIGURE 7:**
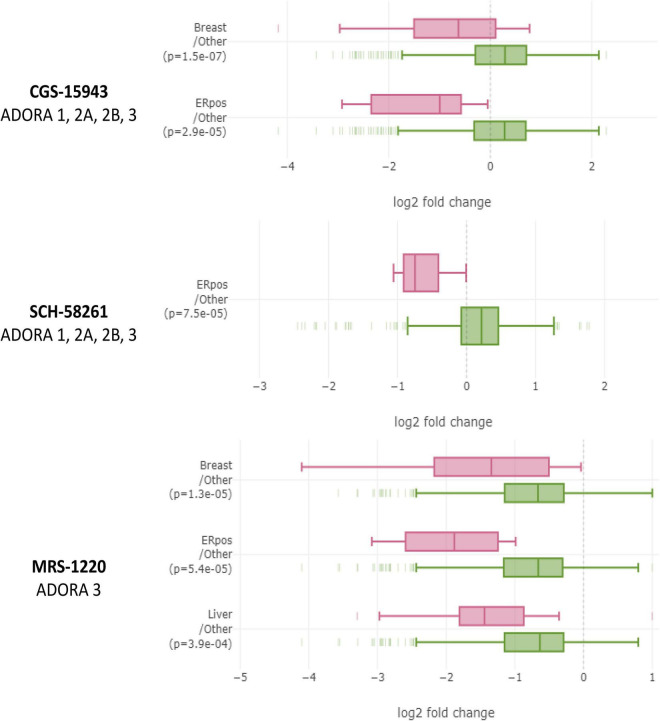
Breast cancer cell lines, especially ER^+^ ones, are uniquely sensitive to non-xanthine adenosine receptor antagonists in large-scale PRISM screen performed by The Drug Repurposing Hub. In the Drug Repurposing Hub, numerous different cell lines are barcoded, pooled, and relative barcode frequency is collected after drug treatment. Non-xanthine A3 antagonists CGS-15943, SCH-68261, and MRS-1220 each decreased relative quantities of ER^+^ breast cancer cell lines relative to other cell lines. Data were analyzed on DepMap portal using the limma R statistical package. Significant differences were considered by *p* < 0.0005.

The Drug Repurposing Hub measures differential sensitivity of numerous cell lines to pharmacologic agents (33). We analyzed non-xanthine A3 antagonists CGS-15943, SCH-58261, and MRS-1220 because of their ability to block adenosine receptors without phosphodiesterase inhibition (47). The results strongly supported our hypothesis. Breast cancer cells, especially ER^+^ ones, are uniquely sensitive to these three drugs at 2.5 μM (Figure 7). Furthermore, 22Rv1 cells displayed similar sensitivity as ER^+^ breast cancer cells, though there were too few prostate cancer cell lines to draw conclusions about prostatic cell lines as a whole. It should be noted, that using this technique, there is a limitation in the lack of connection of this data with the metastatic potential and targeting of the cancer. However, given the prevalence of bone metastasis in breast and prostate cancer, coupled with our other results, we generalize that treatment could lead to far-reaching impact. These data suggest that A3R inhibition may be a new therapeutic avenue for the treatment or prevention of ER^+^ breast and prostate cancer bone metastases and further highlights the importance of eADO signaling in cancer.

## Discussion

Metastasis is an inefficient process, and few disseminated cells successfully become overt metastases (48). Bone is a highly vascularized tissue (49) easily accessible by circulating cancer cells. An overwhelming majority of cancer deaths are caused by metastasis (50, 51) and bone is the most common metastatic site for ER^+^ breast and prostate cancers (1, 2). Understanding the factors that prevent most breast and prostate cancer cells from colonizing this new environment and how some cells bypass these barriers is vital for preventing and treating bone metastases, and also determining which tumors may be low risk. Despite advances in bone metastasis treatment, clinical outcomes after bone metastases remain poor (5, 7, 52, 53). Prevention of breast cancer bone metastases by bisphosphonates is a rare example of a drug targeting a potential metastatic site, effectively reducing metastasis there (13).

**FIGURE 8:**
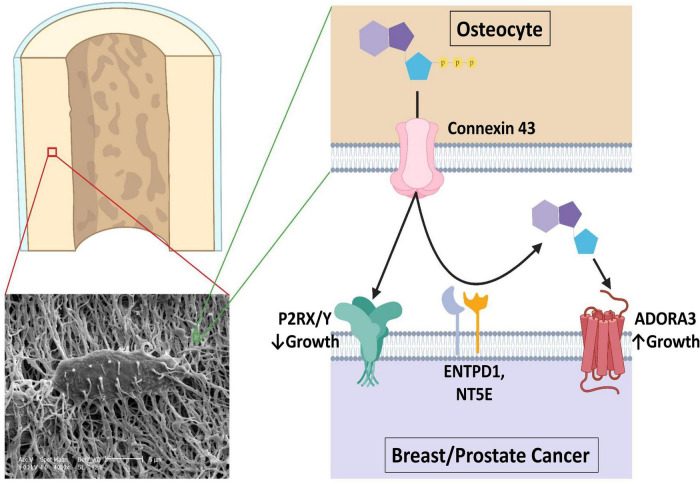
A proposed model of the role of purinergic signaling in breast and prostate cancer bone colonization. An estimated 42 billion human osteocytes reside in a lacuna-canalicular network with an estimated surface area of 215 m2 and an extracellular volume of 24 ml (70). Connexin 43 hemichannel activity is promoted by bisphosphonate treatment and in response to shear stress such as seen in exercise, through which ATP is released that usually inhibits breast and prostate cancer growth in bone through ATP receptor stimulation. However, CD39 and CD73 (encoded by ENTPD1 and NT5E) work in concert to hydrolyze the extracellular ATP in the bone microenvironment to ADO, where it is able to activate A3 receptors and promote growth. Figure was made using BioRender.

Bisphosphonates have long been known to induce apoptosis of osteoclasts (54). We and others have reported that bisphosphonates also promoted osteocytes, the predominant bone cell, to release ATP to the extracellular environment through Cx43 hemichannels and that this decreases triple-negative breast cancer growth in bone (15–17, 55). We further found that eATP signaling inhibits and eADO promotes growth and migration in these cell lines. The present study provides new findings in several ways. We showed that expression of a three-gene expression signature comprising ENTPD1, NT5E, and ADORA3 in primary ER^+^ breast and prostate cancers was correlated with bone metastases. The fact that these genes were much more highly expressed in bone metastases than in other locations or in primary tumors lends further support for their role in metastasizing bone, a tissue rich in Cx43 hemichannels that release ATP. The growth inhibitory effect of the non-hydrolyzable ATP analog ATPγS compared to eATP on 3D cultures of prostate (22Rv1) and ER^+^ breast (MCF-7) directly showed the importance of these cells’ ability to evade their environment from eATP. We also found that A3R inhibition by MRS-1220 inhibits growth in 3D culture of both cell lines and a wide range of ER^+^ breast cancer cell lines. Altogether, our data may support a model shown in Figure 8. Osteocytes release ATP to the bone microenvironment that inhibits colonization of ER^+^ breast and prostate cancers through the activation of one or more ATP receptors. However, in cells that have a greater ability to hydrolyze eATP to eADO through ENTPD1 and NT5E expression, there is less eATP-mediated inhibition. Instead, the generated eADO activates A3 receptor, enabling bone colonization. Future studies should be done, utilizing technology such as siRNA or CRISPR-KO/KD, to determine the direct role of ENTPD1, NTSE, and ADORA3 in cancer cell behavior.

There is a striking difference between breast and prostate cancer bone metastases, with tumors from breast usually displaying an osteolytic, bone destructive phenotype, while tumors from prostate usually adopting an osteoblastic phenotype with increased localized bone density (56). With our data consistent between two very different phenotypes, it is possible that skeletal metastases from other primary tumors share some of the same vulnerabilities and mechanisms.

Bone metastasis is a usually fatal complication that can occur with many cancer types. Unlike other locations, there are treatments that target bone rather than the cancer cells. So far, prophylactic bone metastasis trials have reported mixed results (13, 57, 58). However, these drugs may not be targeted at the right cohort of patients. Because of the long time span in which a metastasis can occur, many available genomic classifiers were designed to predict recurrence (59, 60). These often have limited predictive value for other outcomes. Our data suggest that there may be gene(s) in bone metastasis expression shared between cancers of multiple primary sites. Thus, the ENTPD1/NT5E/ADORA3 signaling axis has the potential to be used as a biomarker or therapeutic target to predict, prevent, or treat bone metastases from multiple sites. Future work should focus on the collection and analysis of this gene signature from primary and bone metastatic cancer sites as well asfrom a broader set of patients.

Inhibiting antibodies against CD39 (encoded by ENTPD1) and CD73 (encoded by NT5E) have recently been developed and are in clinical trials in an immunotherapeutic context (19). Adenosine receptor antagonism, especially of A2A, is also a promising immune stimulator (61, 62). Our data suggests that a separate mechanism inhibiting CD39 and CD73 may be particularly effective in treating or preventing bone metastasis if used in combination with an A3 inhibitor. These classes of drugs may have further enhancement in combination with bisphosphonate treatment.

Preventing bone metastasis may also reduce metastases to other locations. In the overwhelming majority of patients with metastatic ER^+^ breast cancer, the initial presentation includes bone (35), and most patients who first present with skeletal metastases later develop metastases at other locations (63). Genetic evidence of bone metastases seeding other metastases has been found for both breast (64) and prostate (65–67) cancer. The bone microenvironment has been shown in experimental models to enhance the plasticity of ER^+^ breast cancer cells (68) and strongly increase the ability of breast and prostate cancer cells to colonize in the lung and other organs from leg tumors (69). Thus, the importance of studying and preventing bone metastasis may be even higher than is currently appreciated.

## Conclusion

A 3-gene signature composed of ENTPD1, NT5E, and ADORA3 is associated with a greater chance of bone metastasis in ER^+^ breast and prostate cancers. These genes are more highly expressed in bone metastases than in other metastases or primary tumors. These genes encode enzymes that hydrolyze eATP to eADO, and an eADO receptor. In 3D culture, eATP decreased spheroid sizes of MCF-7 and 22Rv1 ER^+^ breast and prostate cancer cell lines. ATPγS, which is resistant to hydrolysis, further decreased spheroid sizes. These cell lines are sensitive to MRS-1220, a specific A3R inhibitor. ER^+^ breast cancer cell lines are sensitive to adenosine receptor inhibition.

## Data availability statement

The original contributions presented in this study are included in the article/Supplementary material, further inquiries can be directed to the corresponding author.

## Author contributions

DS, JJ, L-ZS, and VJ: conceptualization. DS, FA, JB, and KF: methodology and validation. DS and KF: software. DS: formal analysis, investigation, data curation, and writing—original draft preparation. JJ and L-ZS: resources. FA, L-ZS, KF, VJ, and JJ: writing—review and editing. JJ: project administration and funding acquisition. All authors read and agreed to the published version of the manuscript.
