# Supplementary material for: Association of adenosine signaling gene signature with estrogen receptor-positive breast and prostate cancer bone metastasis
Source: Front Med (Lausanne). 2022 Sep 15;9:965429. doi: 10.3389/fmed.2022.965429 (PMC9520286; doi:10.3389/fmed.2022.965429)
Supplement: Supplementary file 1 [file Data_Sheet_1.PDF]

frontiers

Supplementary Material

Dataset	Type	Characteristics
GSE74685	Microarray	human, metastatic prostate cancer samples
GSE14020	Microarray	human, breast cancer metastasis samples
GSE32269	Microarray	human, localized and bone metastatic prostate cancer samples
GSE47561	Microarray	human, renormalized breast cancer related samples
GSE124647	Microarray	human, biopsies of metastatic HR+/HER- breast cancer
TCGA BRCA	Gene set	human, breast cancer database
TCGA PRAD	Gene set	human, prostate cancer database
DKFZ	Gene set	human, prostate cancer database

Table S1. Description of datasets utilized.

Gene	log2(FC)	p value
P2RY2	-0.247	0.21677
P2RY6	-0.237	0.17539
P2RY14	-0.226	0.18967
P2RX4	-0.164	0.39994
ADORA1	-0.117	0.46132
P2RX2	-0.089	0.59293
P2RY13	-0.074	0.62806
P2RX7	-0.052	0.74989
P2RX3	-0.048	0.23121
P2RX6	-0.019	0.78163
P2RX6	-0.013	0.83560
P2RX2	-0.013	0.90782
P2RX5	-0.010	0.92257
P2RX1	-0.003	0.96828
ADORA1	0.032	0.67143
P2RY1	0.051	0.19588
P2RY4	0.067	0.33806
ADORA2B	0.346	0.10423

Table S2. Purinergic receptor expression in breast cancer bone metastases compared to metastases to other sites.

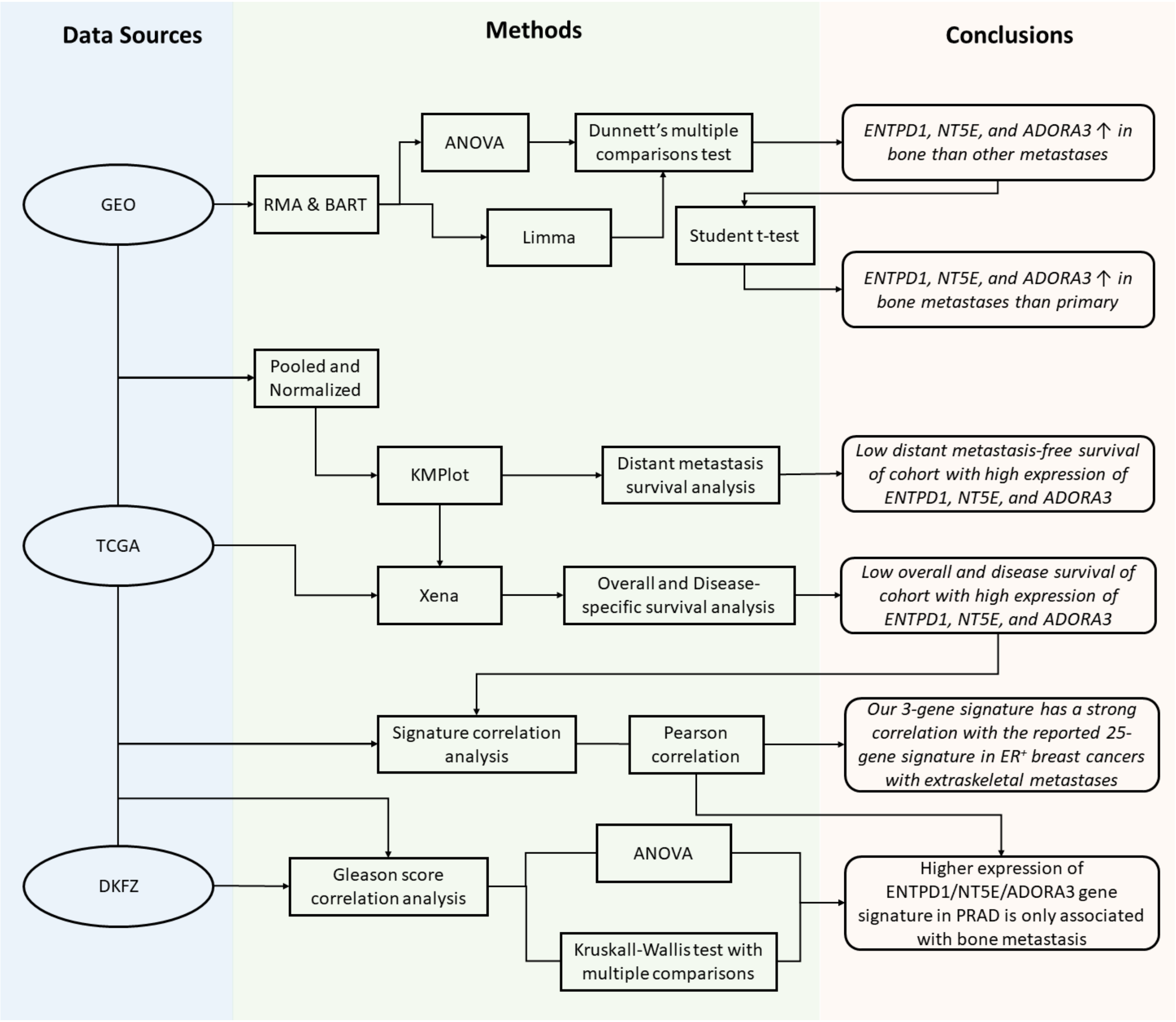

Supplementary Figure 1. Workflow for Analysis Conducted.

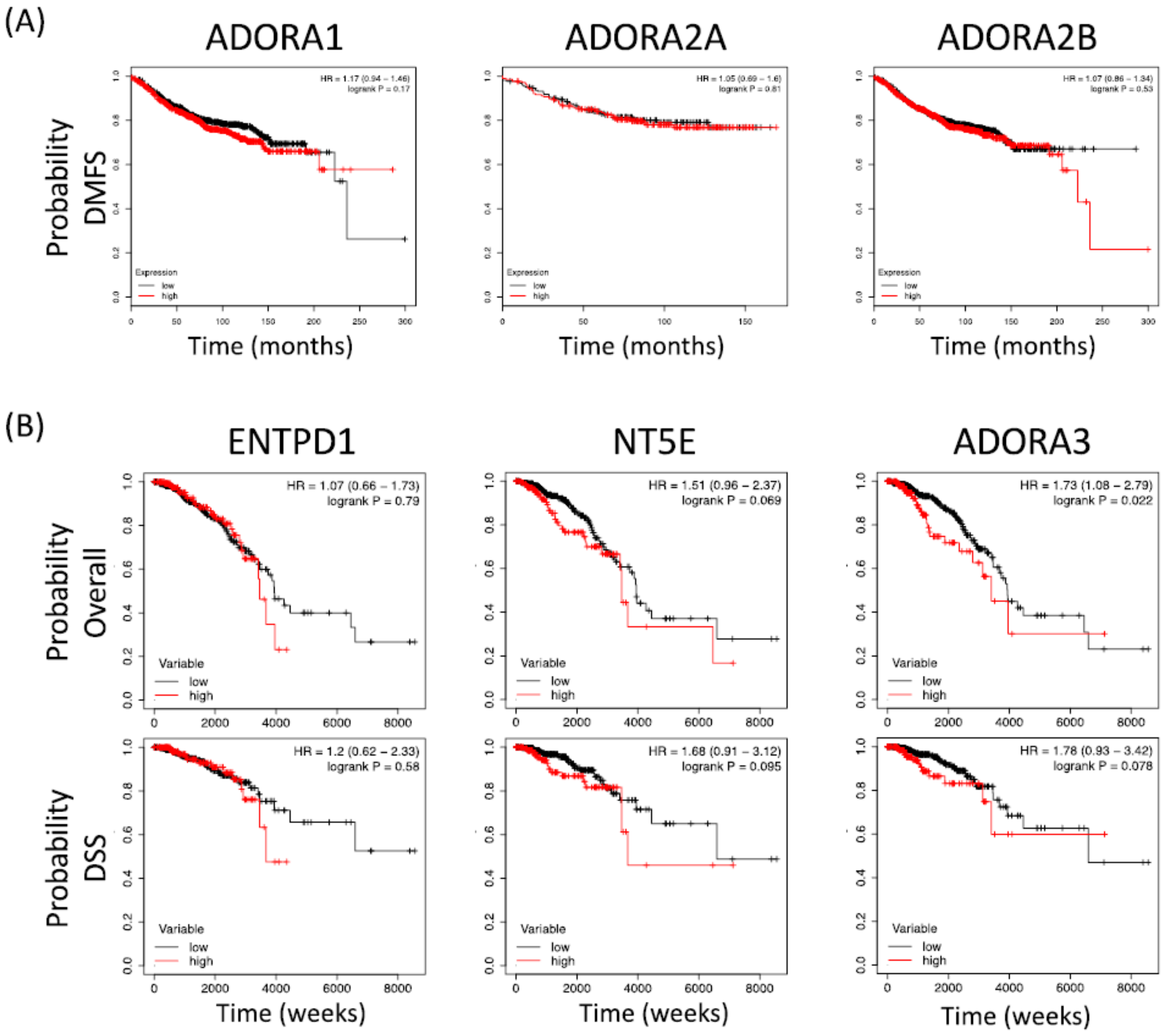

Supplementary Figure 2. Overall and disease-free survival in situations related to Fig. 2B. Survival curves and logrank tests were used investigate how ENTPD1, NT5E, and adenosine receptor expression are correlated with outcomes in a variety of situations. Analyses were performed as in Fig. 2. **(A)** Expression of ADORA1, ADORA2A, and ADORA2B are not associated with distant metastasis-free survival in primary ER+ breast cancers. Individually, the correlation between ADORA3 and overall survival is the only significant relationship. **(B)** In ER+ breast cancer, ENTPD1 and NT5E expression are individually not associated with disease-specific or overall survival. High ADORA3 expression is associated with lower overall, but not disease-specific survival.

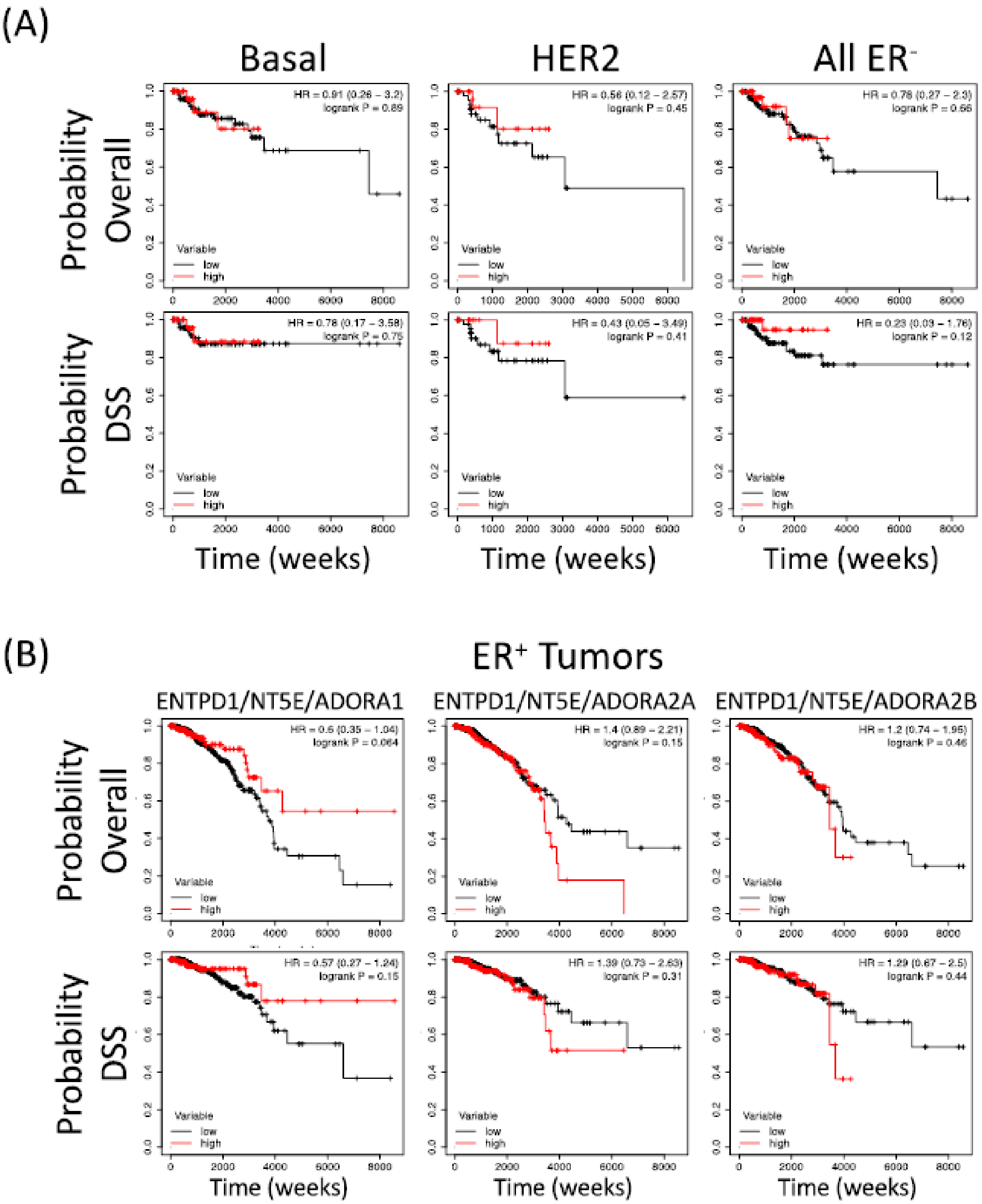

Supplementary Figure 3. Overall and disease-free survival in other breast cancer subtypes and difference gene signatures in ER^{+} breast cancer. (A) The ENTPD1/NT5E/ADORA3 expression signature is not associated with overall or disease-specific survival in basal or HER2 molecular breast cancer subtypes or in ER^{-} tumors in the TCGA BRCA cohort. (**B**) Expression signatures combining ENTPD1, NT5E, and other adenosine receptors in the ER^{+} TCGA cohort are not associated with overall or disease-specific survival.

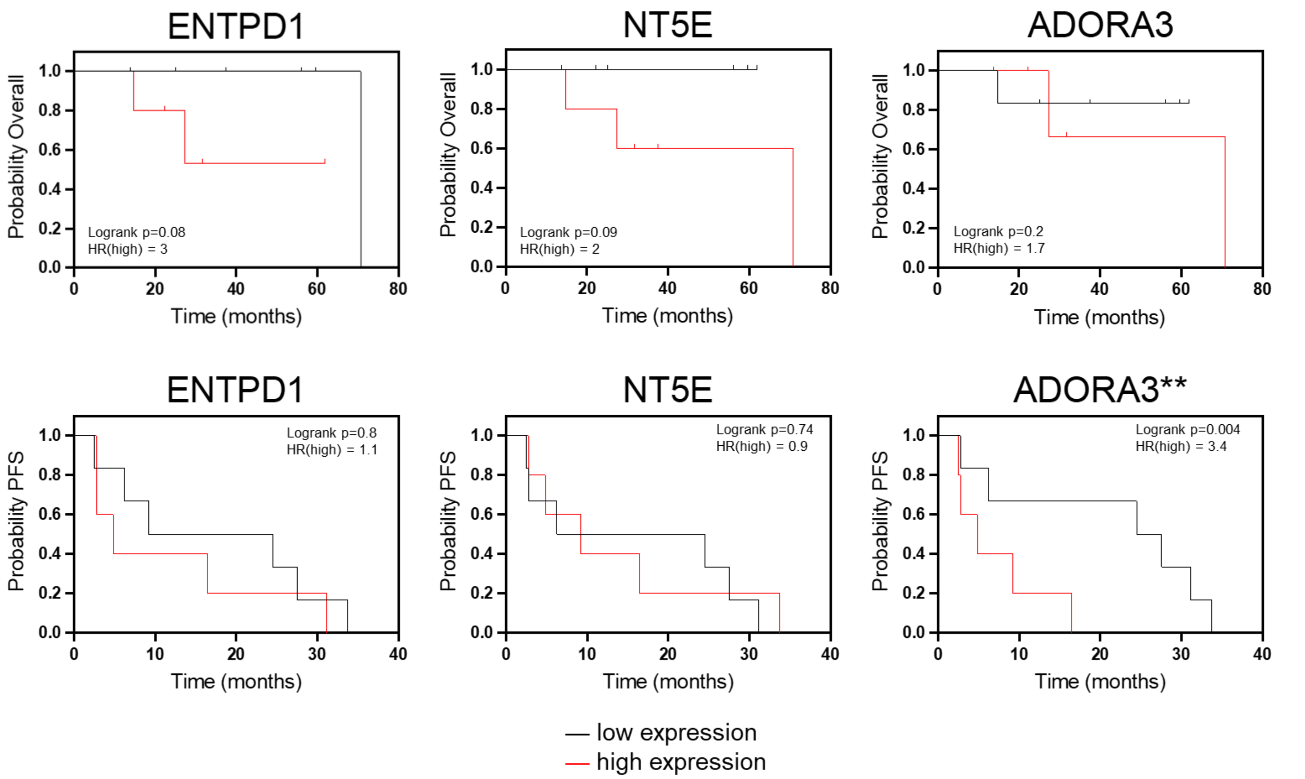

Supplementary Figure 4. Overall and progression-free survival of patients with bone metastases. Analysis was performed similar to Fig. 3B using individual gene expression rather than signature. Samples were taken from bone metastases of patients with ER^+ breast cancer. The ADORA3 high-expression group had significantly longer progression-free survival, but there were no significant differences in the other analyses.
